# Comparative evaluation of high-flow nasal cannula (HFNC) and conventional oxygen therapy (COT) in infants after non-cardiac surgery: Study protocol of a randomized controlled trial

**DOI:** 10.1371/journal.pone.0314782

**Published:** 2025-01-08

**Authors:** Diwei Zhang, Tianqing Gong, Qinghua Huang, Qianqian Zhang, Kai Liu, Jia Li, Hai Yu, Yu Cui

**Affiliations:** 1 Department of Anesthesiology, Chengdu Women’s and Children’s Central Hospital, School of Medicine, University of Electronic Science and Technology of China, Chengdu, China; 2 Department of Anesthesiology, West China Hospital, Sichuan University, Chengdu, China; Asan Medical Center, University of Ulsan College of Medicine, REPUBLIC OF KOREA

## Abstract

**Background:**

Literature regarding the advantages of HFNC in infants for ensuring oxygen supply after non-cardiac surgery is insufficient. The purpose of our study is to compare COT vs. HFNC on postoperative outcomes in infants undergoing non-cardiac surgery. We hypothesize that prophylactic use of HFNC after non-cardiac surgery in infants would reduce the incidence of post-anesthesia hypoxemia and could also be adapted as first-line oxygen therapy after non-cardiac surgery.

**Methods:**

This is a superior, single-blind, randomized controlled study. A total of 394 infants undergoing general anesthesia will be randomly assigned to accept COT or HFNC in a 1:1 ratio. The primary outcome is the rate of desaturation post-extubation. Secondary outcomes include the rate of mild upper airway obstruction, the rate of severe respiratory depression, the rate of transfer to PICU, duration of oxygen therapy, length of PACU stay, the time to reach full enteral feeding, and postoperative adverse events, including nasal injury, agitation, vomiting, and unplanned secondary surgery related to the initial surgery.

**Discussion:**

This is the first randomized controlled trial to explore the advantages of HFNC in infants to ensure oxygen supply after non-cardiac surgery. If favorable evidence is obtained, HFNC could be adopted as first-line oxygen therapy for infants following non-cardiac surgery.

**Trial registration:**

The trial was registered at https://www.chictr.org.cn (Registration number: ChiCTR2400081600, Date: March 6, 2024).

## Background

Postoperative hypoxemia after general anesthesia is very common that can affect from 4% to 57% of patients in some cohorts [1–6]. To our knowledge, it occurs more often in young infants than in older children or adults. The risk factors associated with postoperative hypoxemia in children include age, body weight, surgical type, anesthesia methods, insufficient oxygen supplement during transportation, and comorbidity (i.e., obstructive sleep apnea syndrome) [2,6].

Young infants, with premature lungs, or who are even hemodynamically unstable, may increase the risk of postoperative hypoxemia. Traditionally, conventional oxygen therapy (COT), such as a standard nasal cannula, was routinely used in the post-anesthesia care unit (PACU) to decrease postoperative pulmonary complications. However, for infants, the standard nasal cannula necessitates limiting the maximum flow to 2 L/min [7]. Even with humidification, flow rates > 6L/min are not recommended with the standard nasal cannula due to limitations inherent in bubble humidification [8]. Thus, despite administering oxygen supplementation, the incidence of postoperative hypoxemia in newborns and infants was still as high as 3.8% [2]. Therefore, it is urgent to explore strategies to reduce the incidence of postoperative hypoxemia.

The use of “high flow nasal cannula” (HFNC), a relatively new mode of respiratory support, is increasingly being used in infants [9]. It is mainly applied in the field of the intensive care unit (ICU) and after extubation in children undergoing cardiac surgery. The advantages of using HFNC after cardiac surgery are still underdetermined. A meta-analysis reported that compared with COT, HFNC significantly increased PaO_2_ and the ratio of PaO_2_ to FiO_2_, and decreased PaCO_2_ [10] after cardiac surgery; whereas, Kuitunen et al. did not find clear evidence of a difference in reintubation rates and length of PICU stay between different noninvasive ventilation strategies [11]. In our hospital, more than 10,000 pediatric non-cardiac surgeries are performed per year, of which 10% are infants. For this vulnerable group, their oxygen reserve is poor, and they are more prone to developing desaturation after surgery. Whether HFNC is more effective than COT in ensuring oxygen supply after non-cardiac surgery has never been investigated. Thus, we intend to conduct a randomized clinical trial to investigate the advantages of HFNC in lowering the risk of desaturation after general anesthesia in infants. We hypothesized that prophylactic use of HFNC after non-cardiac surgery in infants would reduce the incidence of post-anesthesia hypoxemia.

## Methods/Design

### Study design, setting, ethics, and trial registration

This study is a randomized clinical trial in a tertiary teaching hospital. It will be conducted following the principles of the Declaration of Helsinki. The study protocol has been structured in accordance with the SPIRIT 2013 Statement and CONSORT 2010 guidelines. This trial is approved by the Institutional Review Board of Chengdu Women’s and Children’s Central Hospital (IRB approved number: 2024 [17], approval date: January 1^st^, 2024, estimated duration of the trial: March 2024 to Feb 2025). This study is also registered at https://www.chictr.org.cn (registration number: ChiCTR2400081600, date: March 6, 2024) before the first patient enrollment. The protocol that was approved by the ethics committee is attached as a S2 File.

### Patient inclusion and exclusion criteria

**Inclusion criteria.** Infants under the age of 12 months who are scheduled to undergo elective non-cardiac surgery;

- American Society of Anesthesiologists (ASA) classificationⅠ~Ⅱ;
- Patients receive general anesthesia with an endotracheal tube;
- The endotracheal tube is removed in the operating room or post-anesthesia care unit (PACU).

### Exclusion criteria

- Patients with cardiac/hepatic/renal dysfunction;
- Patients with pulmonary dysfunction or congenital airway malformation, including bronchopulmonary dysplasia, tracheal or subglottic stenosis, respiratory distress syndrome, active upper respiratory infection, asthma, and definitively diagnosed syndromes associated with anticipated difficult airway management;
- Patients who have undergone intubation before surgery;
- Patients with any allergic history of anesthetic;
- Patients with SpO_2_<95% in room air before anesthesia;
- Patients admitted to ICU with endotracheal tube;
- Patients whose guardians refuse to participate.

### Enrollment of participants and allocation

#### Consent to participate, enrollment of participants

Before surgery, one of the investigators who has been trained for the study procedures will screen potential candidates, according to the inclusion and exclusion criteria. Their parent, guardian, or authorized surrogate will be informed of the benefits, drawbacks, and possible risks of participating in the current study. Written informed consent from a parent or guardian or authorized surrogate is required before enrollment.

### Allocation, randomization, and interventions

**Fig 1:**
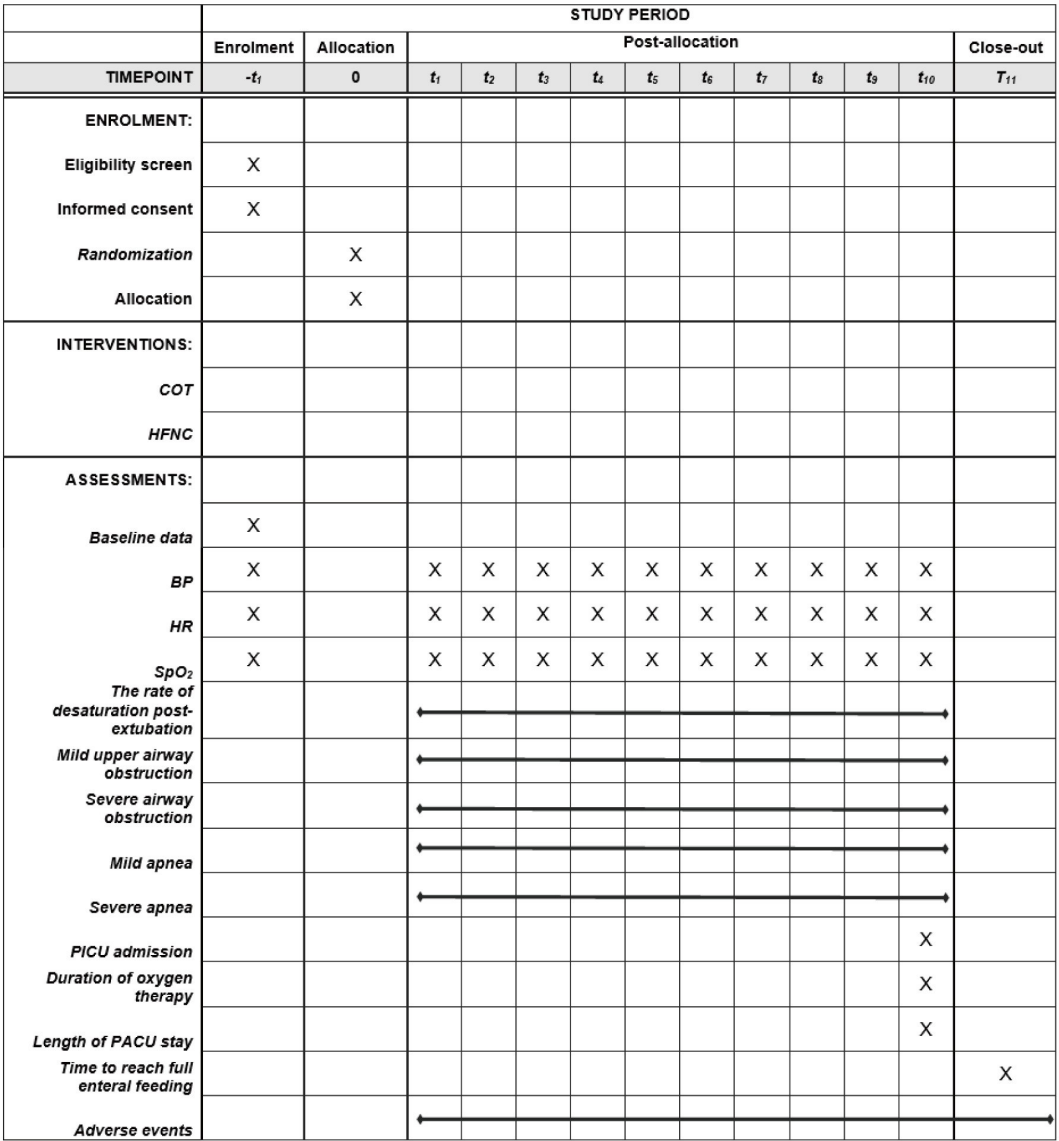
The schedule of enrolment, interventions, and assessments (SPIRIT 2013).

**Fig 2:**
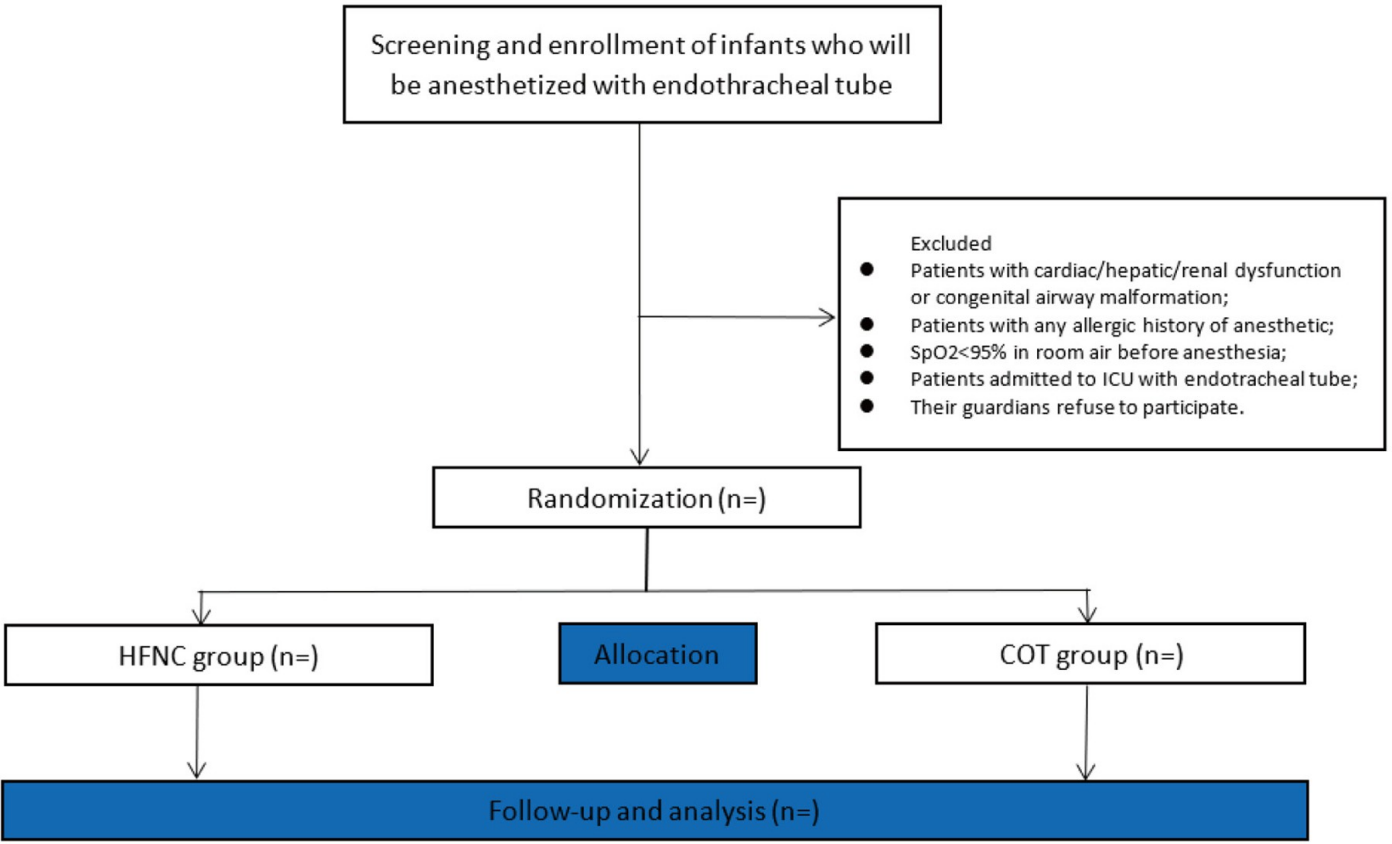
The study flowchart.

This is a randomized, single-blind controlled clinical trial. A computer-generated random number sequence is generated by a researcher who will not participate in any other section of this clinical trial and distributes the allocated opaque envelope. The envelope will be delivered directly to the anesthesiologist who is responsible for the postoperative care of patients. The anesthesiologist working in PACU during our study period will be experienced (≥ 5 years in pediatric anesthesia). A total of 384 eligible patients will be recruited and randomly assigned into either the COT group or the HFNC group in a 1:1 ratio. The investigator responsible for collecting the data cannot be blinded during the study, since the appearance of HFNC equipment and that of COT have obvious differences. However, the investigator will not be involved in clinical decisions and the researcher analyzing the data will be blinded to the groups’ assignment. This study is in line with SPIRIT reporting guidelines, which is attached as an online S1 File. Fig 1 is the SPIRIT Schedule of enrolment, interventions, and assessments. And, the study flowchart is presented in Fig 2.

### Interventions

#### Anesthesia management

To reduce separation anxiety, midazolam (1 mg/kg) will be given intravenously before the infants enter the operating room. Then, the patient will be continuously monitored, including electrocardiogram (ECG), SpO_2_, and non-invasive blood pressure. The choice of anesthetic agent for induction and maintenance is determined by the anesthesiologist. A bolus dose of analgesics and muscle relaxants is permitted to be given during the surgery if necessary, which is determined by the anesthesiologist on duty.

#### Postoperative care

In our routine practice, to make the operating room work more efficiently, mechanical ventilation continues after the patient is transferred to PACU until they meet the extubation requirements. For pediatric patients after surgery, the endotracheal tube is removed when the patient meets the following conditions. (1) a tidal volume >5 ml/kg; (2) The patient’s full awakening was assessed through clinical signs, including conjugate gaze, facial grimace, eye-opening and purposeful movements [12]; alternatively, the patient is under deep anesthesia, with reversal of the neuromuscular blockade confirmed by a train-of-four (TOF) ratio exceeding 0.9 [13].

After extubation, infants who are allocated to the COT group, as routine practice, will receive humidified oxygen at 2~5 L/min through a nasal cannula (2L/min initially, and the highest is 5 L/min), while patients in the HFNC group receive oxygen at 1~2 L/kg/min through the HFNC system (Mindray, Shenzhen, China) depending on the patient’s tolerance [14]. In the HFNC group, flow is initially set at 1L/kg/min and the flow is titrated upwards in 0.5L/kg/min steps, at a maximum of 2L/kg/min. The initial fraction of inspired oxygen (Fio_2_) is 100%. The temperature is set to 37°C with a humidifier. Two types of nasal cannula are selected according to the infant’s weight and nare size. Nasal cannulas are 50% smaller than the infant’s nares to avoid excessive airway pressures [14]. The target SpO2 in the COT group and the HFNC group is maintained ≥ 95% [12]. The decision to discontinue oxygen therapy is at the discretion of the anesthesiologist who is responsible for patients’ care in PACU. Criteria for reintubation include any of the following: respiratory or cardiac arrest, respiratory pauses with loss of consciousness or gasping for air, psychomotor agitation inadequately controlled by sedation, massive aspiration, the persistent inability to remove respiratory secretions, heart rate lower with loss of alertness, and severe hemodynamic instability unresponsive to fluids and vasoactive drugs, and patients who developed persistent post-extubation respiratory failure were also reintubated [15]. Postoperative infants should stay in the PACU for at least 30 minutes.

#### Criteria for discontinuing or modifying allocated interventions

Patients can quit the study at any time during the procedure, regardless of any reason. The research can discontinue infants’ participation if they have serious adverse events.

### Data collection

The following data will be collected. To guarantee the study quality, all investigators involved in the current study will be trained before the first case is enrolled. TG will design the random sequence which will be generated on the random number generator. The trained investigator (DZ) will collect postoperative data in PACU and complete the 24-hour follow-up, and the data will be double-checked by the third investigator (QH). To avoid bias, the statistical analyses will be conducted by the corresponding author (YC) who will not participate in data collection and will be blinded to the patient’s allocation.

- *Demographic data*: age, gestational age, whether the infant was born preterm, weight, gender, ASA status, clinical diagnosis, past medical history, surgery history, allergic history, surgical index, and the baseline of vital signs, including SpO_2_, heart rates (HR), and mean blood pressure (MBP).
- *Intraoperative data*: surgical duration, anesthesia duration, intubation time, tube size, anesthetics consumption, muscle relaxant consumption, and muscle relaxant antagonist consumption.
- *Postoperative data*: The SpO_2_, MAP and HR will be collected 1min before extubation (T1), at the time point of extubation (T2), 1 min after tracheal extubation (T3), 5 min after tracheal extubation (T4), 10 min after tracheal extubation (T5), 15 min after tracheal extubation (T6), 20 min after tracheal extubation (T7), 25 min after tracheal extubation (T8), 30 min after tracheal extubation (T9), and at the time point of discharge from PACU (T10). The incidence of mild and severe respiratory depression, the requirement for tracheal reintubation, and the need to transfer to the Pediatric Intensive Care Unit (PICU) will be documented from the patient admitted into PACU to discharge from PACU. The duration of oxygen therapy will be recorded. The patient will be followed at 24 hours after surgery, and time to reach full enteral feeding will be collected. Besides, the adverse events, such as nasal injury, agitation, vomiting, and unplanned secondary surgery related to this surgery, will be recorded.

### Data management and confidentiality

The CRF is designed by DZ according to our protocol and will be deposited at the Department of Anesthesia for 10 years securely. Only the members of study team will have access to stored data at a reasonable request. Any missing or errors in the data will be summarized along with detailed descriptions and will be queried by checking the original CRF and electronic medical records. Once the study is completed, the key to safety will be preserved by the principal investigator (YC) who is the corresponding author. After the completion of the clinical trial, the electronic data will be organized and publicly released on the Dryad database (https://datadryad.org). The identification of the enrolled infants will not be revealed in publications.

### Data monitoring

The data monitoring committee will not be established since this is a small-scale study conducted at a single center. When serious adverse events associated with our intervention occur during the procedure, the principal investigator has the right to review those results, unblind the study, and even make a final decision to terminate the trial. Any modification to the protocol will be discussed and agreed to by the investigators and will be subsequently submitted to the IRB for approval. The new protocol will also be updated at the Chinese Registry of Clinical Trials.

### Outcome measures

The primary outcome is the rate of desaturation post-extubation, which is defined as SpO_2_ < 95% after extubation. SpO_2_ will be monitored using a non-invasive blood oxygen saturation sensor. The rate of desaturation and its duration will be recorded. The duration of desaturation is defined as the period between the time point when SpO_2_< 95% to recovery ≥ 95%.

The secondary outcomes are collected as follows:

- Mild upper airway obstruction refers to the airway obstruction being immediately improved by changing the posture.
- Severe airway obstruction, which indicates the patient needs airway management (including laryngeal mask ventilation, tracheal reintubation, or placement of other airway devices) to maintain SpO_2_ ≥ 95%.
- Mild apnea, defined as apnea that resolves spontaneously or with tactile stimulation.
- Severe apnea, defined as apnea requiring positive pressure ventilation.
- The determination of transfer to PICU is judged by the attending physician who takes responsibility for PACU.
- The duration of oxygen therapy was defined as the period from the time point of extubation to the time point that the patients can maintain SpO_2_ ≥ 95% without oxygen supplement.
- Length of PACU stay refers to the duration from extubation to discharge from PACU.
- The time to reach full enteral feeding means the time of first eating after surgery, including a light drink.
- The adverse events include nasal injury, agitation, vomiting, and unplanned secondary surgery related to this surgery.

### Safety, adverse event reporting, and harms

Although this is a prospective interventional study, the oxygen therapy that will be used postoperatively is non-invasive, regardless of the group allocation. Thus, the safety of patients can be guaranteed. Serious adverse events such as death, cardiac arrest, or disability will be treated in a timely and appropriate manner. All serious adverse events should be reported to the project leader, the institute, and the ethics committee within 24 hours.

### Sample size

The current study is powered by the primary outcome. The sample size in this prospective study was calculated according to the authors’ pilot study with desaturation rates of 20.5% and 10% in the COT group and the HFNC group, respectively, with a standard deviation of 0.2. To achieve an 80% statistical power with an error of 0.05, the number of patients was calculated to be 174 for each group. Considering 10% of the sample drop, 192 patients were enrolled in each group. For sample size calculation, the R studio software, version 4.2.2 was used.

### Statistical analysis

The R studio software, version 4.2.2, will be used for statistical analysis. Data will be presented as numbers (percentage), mean±standard deviation, or median (minimum-maximum) as appropriate. The t-test or Mann-Whitney U test will be performed for continuous variables between groups according to the distribution of data (tested by the Kolmogorov-Smirnov test). The x^2^ test or Fisher’s exact test will be used to compare the differences in categorical data. A value of p < 0.05 indicates statistical significance. A subgroup analysis will be conducted based on whether the postconceptional age is less than 60 weeks.

## Discussion

This is a prospective randomized controlled clinical trial to investigate the advantages of HFNC in lowering the risk of desaturation after general anesthesia in infants. Although it is impossible to blind the investigator collecting the data, we try our best to perform the randomization and allocation concealment in the current study. HFNC is a respiratory device that can deliver warm and humidified gas at a higher flow rate than the patient’s inspiratory flow rate, and it is increasingly utilized in critically ill infants [8].

Through the literature review, we find that most studies about the utilization of HFNC in infants focus on preventing reintubation after cardiac surgery or treating respiratory failure [14,16–18]. As we know, the number of infants undergoing cardiac surgery is far less than that of infants undergoing non-cardiac surgery. Thus, prevention of desaturation after non-cardiac surgery in infants is equally essential, and COT cannot well resolve this problem. A meta-analysis found that compared to COT, a large reduction in treated failure with HFNC (11.2% less) was observed [9]. We assume that HFNC may be a better alternative to COT and improve patient outcomes. The results may help to improve the postoperative outcomes for general anesthesia in infants undergoing non-cardiac surgery.

## Trial status

The current protocol is version 3.0, dated December 01, 2023. This trial was registered with the Chinese Clinical Trial Registry (http://www.chictr.org.cn) on March 06, 2024 (Registration no. ChiCTR2100042915). Patient recruitment began on March 08, 2024. The trial is expected to end in May 2025. To date, 116 patients have been enrolled.

## Supporting information

S1 File SPIRIT reporting guidelines. (PDF)

S2 File The protocol that was approved by the ethics committee. (DOCX)
