# Supplementary material for: Comparative evaluation of high-flow nasal cannula (HFNC) and conventional oxygen therapy (COT) in infants after non-cardiac surgery: Study protocol of a randomized controlled trial
Source: PLoS One. 2025 Jan 8;20(1):e0314782. doi: 10.1371/journal.pone.0314782 (PMC11709249; doi:10.1371/journal.pone.0314782)
Supplement: S2 File — (DOCX) [file pone.0314782.s002.docx]

**临床研究方案**

**（方案版本号：**V1.0，**版本日期：**2024年1月22日）

**项目名称：比较高流量吸氧与常规吸氧在预防婴儿全麻手术后低氧血症的差异: 一项前瞻性随机对照临床研究**

**项目类型：干预性实验**

**项目来源单位：成都市妇女儿童医院**

**项目参加单位：成都市妇女儿童医院**

**项目负责人（/主要研究者）：崔宇**

**承担科室：麻醉科**

**目录**

[**1 研究背景 4**](#_Toc30051)

[**2 研究目的 4**](#_Toc8193)

[2.1 主要研究目的 4](#_Toc28809)

[2.2 次要研究目的 4](#_Toc12281)

[**3 研究设计 4**](#_Toc14339)

[3.1样本量计算 4](#_Toc136)

[3.2 随机分组 4](#_Toc10748)

[3.3对照 4](#_Toc30153)

[3.4 盲法及揭盲 4](#_Toc1625)

[**4 研究对象 5**](#_Toc21363)

[4.1 诊断标准 5](#_Toc8115)

[4.2 纳入标准 5](#_Toc20600)

[4.3 排除标准 5](#_Toc11530)

[4.4 退出标准 5](#_Toc31843)

[4.5 脱落与处理标准 5](#_Toc2589)

[4.6 剔除标准 5](#_Toc11468)

[**5治疗方案 6**](#_Toc25494)

[**6访视观察项目 6**](#_Toc7014)

[6.1 一般项目 6](#_Toc30046)

[6.2 诊断和疗效性指标 6](#_Toc24939)

[6.3 安全性指标 6](#_Toc29916)

[6.4 访视检查流程及注意事项 6](#_Toc9071)

[6.5 患者依从性及退出 7](#_Toc19953)

[**7疗效判定标准 7**](#_Toc6381)

[7.1 症状或体征疗效评定 7](#_Toc20705)

[7.2综合疗效评定 7](#_Toc13571)

[**8 不良事件的处理和报告 7**](#_Toc8338)

[8.1 不良事件的定义 7](#_Toc8756)

[8.2 严重程度评估 7](#_Toc27597)

[8.3 相关性判断 8](#_Toc4404)

[8.4 不良事件的处理 9](#_Toc4443)

[8.5 严重不良事件的记录与报告 10](#_Toc6912)

[**9 质量控制与质量保证 11**](#_Toc14547)

[9.1 质量控制 11](#_Toc20013)

[9.2 质量保证 11](#_Toc10430)

[**10 数据处理及保存 11**](#_Toc31393)

[**11 统计分析 11**](#_Toc3042)

[**12 统计分析方法 12**](#_Toc81)

[12.1统计描述 12](#_Toc13217)

[12.2统计推断 13](#_Toc3142)

[12.3 统计分析计划 13](#_Toc32072)

[**13 伦理学原则 14**](#_Toc351)

[13.1 伦理委员会审核 14](#_Toc22516)

[13.2 知情同意 14](#_Toc13076)

[13.3受试者的信息保密 15](#_Toc11976)

[**14 方案的修改 16**](#_Toc22367)

[**15 试验组织架构 16**](#_Toc431)

[**16 保密及数据安全 16**](#_Toc28917)

[**17 保险和赔偿 16**](#_Toc2356)

[**18知识产权 16**](#_Toc15407)

[**19 参考文献 （请附上相关文献） 16**](#_Toc26184)

**方案摘要**

于2024年3月10日至2026年3月10日期间在成都市妇女儿童中心医院的主手术室进行。384名小于1岁的患儿将被纳入这项研究。研究小组将在术前评估患者，并获得每位候选人家属的书面知情同意。将从病历中收集人口统计数据和体格检查。将患者随机分为传统常规吸氧组和经鼻高流量吸氧组。在麻醉前立即使用计算机生成的随机编码，使用密封的不透明信封进行随机分组。考虑到研究干预的性质，完全盲法是不可能的。然而，对参与术后评估的研究者对组的分配是不知情的。所有患者术前禁食固体食物8小时以上，禁糖水2小时。在手术室中，连续监测心电图、脉搏血氧测量、无创血压和平均动脉压。所有患者均采用全身麻醉，手术结束后送入麻醉后监护室复苏后拔出气管导管常规氧疗或经鼻高流量吸氧。分时段记录患儿心率、SPO2。

结果

主要结果是术后发生吸氧后SpO2 ≤ 95%的患儿的数量

可以通过改变患儿睡姿或者轻抬下颌缓解症状的轻度上呼吸道梗阻，发生严重呼吸抑制，包括采用口咽通气道、喉罩，才能缓解，术后再次气管插管比例，术后非计划转入ICU比例，术后吸氧时间，术后恢复室停留时间，术后进食时间，鼻黏膜损伤患儿的数量被评估为次要结果。

**缩略语表**

不涉及

**1 研究背景**

全麻术后低氧血症非常常见，发生率为4%~ 57%。据我们所知，相比于较大的儿童或成人，低氧血症更常发生在年幼的婴儿。儿童术后低氧血症的相关危险因素包括年龄、体重、手术类型、麻醉方式、转运过程中给氧不足和患儿本身合并症(如：阻塞性睡眠呼吸暂停综合征)。

婴儿，肺发育不成熟，甚至血液动力学不稳定，这些因素可能会增加术后低氧血症的风险。传统上，常规氧疗（COT）在麻醉后监护室（PACU）常规使用，以减少术后肺部并发症。然而，尽管进行了常规吸氧，新生儿和婴儿术后低氧血症的发生率仍高达3.8%。因此，迫切需要探索降低低氧血症发生率的策略。

经鼻高流量吸氧（HFNC）是一种相对较新的呼吸支持模式，越来越多地用于婴儿。它主要应用于重症监护室（ICU）和儿童心脏手术拔管后，然而心脏手术后的优势仍有待确定。一项荟萃分析报告称，与COT相比，HFNC在心脏手术后显著增加了PaO2和氧合指数，并降低了PaCO2；然而，Kuitunen等人没有发现明确的证据表明不同无创通气策略之间的再插管率和PICU停留时间存在差异。在我们医院，每年进行1万多例儿科非心脏手术，其中10%是婴儿。对于婴儿来说，他们的氧气储备很差，手术后更容易出现低氧饱和度现象。

因此，我们打算进行一项随机临床试验，研究HFNC在降低婴儿全身麻醉后氧合降低风险方面的优势。我们假设，在婴儿非心脏手术后预防性使用HFNC可以降低麻醉后低氧血症的发生率，也可以作为非心脏手术的一线氧气治疗。

**2 研究目的**

研究HFNC在降低婴儿全身麻醉后氧合降低风险方面的优势

**3 试验设计**

前瞻性随机对照临床研究

**3.1样本量计算**

目前的样本含量计算以主要结果为依据。本前瞻性研究中的样本量是根据作者的初步研究计算的，COT组和HFNC组的去饱和率分别为20.5%和10%，标准偏差为0.2。为了在误差为0.05的情况下实现80%的统计功效，计算出每组的患者人数为174人。考虑到10%的样本脱落，计划每组招募192名患者。

**3.2 随机分组**

将患者随机分为传统常规吸氧组和经鼻高流量吸氧组。在麻醉前立即使用计算机生成的随机编码，使用密封的不透明信封进行随机分组。

**3.3对照**

使用常规吸氧。

**3.4 盲法及揭盲**

本研究无法实施盲法。然而，对参与术后评估的研究者对组的分配是不知情的。

**4 研究对象**

**4.1 纳入标准**

患儿年龄<12月；ASA 1-2级；拟行全麻下气管插管的非心脏手术；术后在PACU拔除气管导管；患儿家属自愿加入，并签署书面知情同意书。

**4.2 排除标准**

患儿合并心脏、肺部、肝脏、肾脏疾病；患儿对麻醉药物过敏；术前合并低氧血症；患儿家属拒绝参与。

**4.3 退出标准**

患儿家属中途拒绝参与。

**4.4 脱落与处理标准**

①患儿鼻腔出血无法使用高流量吸氧；患儿家属失访。②脱落的处理：研究者应尽可能与受试者联系，询问理由；因过敏反应、不良事件、治疗无效而退出试验者，研究者应根据实际情况妥善治疗受试者，以保障受试者的权益。

**4.5 剔除标准**

严重违背试验方案，例如受试者被误纳入（违反了纳入/排除标准）等。

**5治疗方案**

试验组：术后经鼻高流量吸氧

对照组：术后常规吸氧

**6访视观察项目**

**6.1 一般项目**

包括生命体征，人口学、家族史，既往史、合并疾病与治疗史、现用药史与试验研究相关的症状和体征等。

**6.2 诊断和疗效性指标**

术后发生吸氧后SpO2 ≤ 95%的患儿的数量；可以通过改变患儿睡姿或者轻抬下颌缓解症状的轻度上呼吸道梗阻；严重呼吸抑制，包括采用口咽通气道、喉罩，才能缓解；术后再次气管插管比例；术后非计划转入ICU比例；术后吸氧时间；术后恢复室停留时间；术后进食时间；鼻黏膜损伤患儿的数量

**6.3 安全性指标**

预期或非预期的不良反应。

**6.4 访视检查流程及注意事项**

| **时期**  **步骤** | | **筛选期** | **治疗期** | **随访期** |
| --- | --- | --- | --- | --- |
|  | 签署知情同意书 |  | × | × |
| 临床资料 | 受试者基本资料 |  |  |  |
| 入选与排除标准 | |  | × | × |
| 受试者访视 | 访视1（拔管时） | × |  |  |
|  | 访视2（拔管后72h） | × |  |  |
|  | 访视3（出院时） | × |  |  |
| 不良事件和器械缺陷记录（非必填） | | × |  |  |
| 方案偏离记录（非必填） | | × |  |  |
| 试验完成情况 | | × | × |  |
| 填写CRF | |  |  |  |

**6.5 患者依从性及退出**

逐步调高吸氧流量，患者不适时停止调高吸氧流量。

**7疗效判定标准**

**7.1 症状或体征疗效评定**

术后发生吸氧后SpO2。

**8 不良事件的处理和报告**

**8.1 不良事件的定义**

不良事件(AE, Adverse Event)：病人或临床研究受试者接受研究实施的治疗后出现的不良医学事件，但并不一定与治疗有因果关系。因此不良事件可以是一种不良的和未预期的体征(例如包括实验室检查异常等)、症状或与治疗有时间相关性的疾病，不考虑是否与有实施的治疗有因果关系。

严重不良事件(SAE，Serious Adverse Event)：是指临床研究过程中发生的需住院治疗、延长住院时间、伤残、影响工作能力、危及生命或死亡、导致先天畸形等事件。

**8.2 严重程度评估**

研究者将参考美国国立癌症研究所不良事件通用术语标准（NCI-CTCAE）（第5.0版）评估不良事件。若指南中对某种不良事件的严重程度未予阐明，研究者可根据1～5级的一般定义并结合医学判断进行评估。不良事件的一般分级：

1级：轻度；无症状或轻微；仅为临床或诊断所见；无需治疗。

2级：中度；需要较小、局部或非侵入性治疗；与年龄相当的工具性日常生活活动受限*。

3级：严重或者医学上有重要意义但不会立即危及生命；导致住院或者延长住院时间；致残；自理性日常生活活动受限**。

4级：危及生命；需要紧急治疗。

5级：与AE相关的死亡。

在级别描述中使用的分号（；）值“或者”。

*工具性日常生活活动指做饭、购买衣物、使用电话、理财等。

**自理性日常生活活动指洗澡、穿脱衣、吃饭、盥洗、用药等，并未卧床不起。

**8.3 相关性判断**

不良事件与实施研究的关系分为肯定有关、很可能有关、可能有关、可能无关、肯定无关。

肯定有关：不良事件符合治疗常见的不良反应类型，其出现与治疗有合理的时间关系，治疗停止后不良事件消失，不良事件与合并治疗（用药或其它治疗操作）或非治疗因素（原发病、并发症、食物、环境等）无关。

很可能有关：不良事件符合治疗常见不良反应类型，其出现与治疗有合理的时间关系，治疗停止后不良事件明显缓解，不良事件与合并治疗（用药或其它治疗操作）或非治疗因素（原发病、并发症、食物、环境等）无关。

可能有关：不良事件符合治疗常见不良反应类型，其出现与治疗有合理的时间关系，治疗停止后不良事件可能缓解，合并治疗或非治疗因素也能解释该不良事件。

可能无关：不良事件与治疗有合理的时间关系，但不符合治疗常见的不良反应类型，治疗停止后不良事件缓解不明显；合并治疗或非治疗因素能解释该不良事件，该类因素去除后不良事件可能缓解。

肯定无关：不良事件与治疗无合理的时间关系，不符合治疗常见的不良反应类型，治疗停止后不良事件无缓解；不良事件与合并治疗或非治疗因素肯定有关，该类因素去除后不良事件明显缓解或消失。

**8.4 不良事件的处理**

**8.4.1 不良事件的记录**

每次访视时，所有观察到或受试者提及的不良事件由研究者记录在原始资料中。记录内容包括以下：事件描述、严重程度、起始日期和终止日期、转归、与临床研究治疗的关系、是否对事件采取了措施、严重程度评价。

**8.4.2治疗和跟踪**

研究者应根据不良事件情况，采取相应措施，以保证受试者健康与安全；其所在专业应保证相关抢救设备和药品的供应，以确保紧急时能够给予相应救治。

不良事件需由研究者跟踪至消失/恢复至基线水平或得知最终结果。跟踪结果亦需记录在原始资料中。

**8.5 严重不良事件的记录与报告**

自受试者签署知情同意书之后到末次随访期间发生的SAE均需要被收集和记录。一旦发生SAE，研究者应尽可能详细的填写,《严重不良事件报告表》，并签名。研究者在获知SAE的24h内报告给临床研究管理部（科教部）、医院伦理委员会。按照临床研究管理部和伦理委员会的相关管理规范执行。

**9 质量控制与质量保证**

**9.1 质量控制**

研究者授权：所有研究者，只有在接受培训并获得主要研究者的授权后，方可进入临床研究并在授权范围内进行操作。

研究过程质控：项目组/专业组质控员、临床研究管理部质量管理员对临床研究过程进行质量控制，指导研究者按照研究方案及临床研究管理的要求进行临床研究。

**9.2 质量保证**

研究者培训：在研究开始前，主要研究者须对所有参与本研究的研究者以及其他与本研究相关的人员进行医院IIT临床研究管理相关管理文件、SOP 和本研究方案相关的培训；以及研究相关的主要疗效测量指标监测标准的规定。研究过程中，如有新的研究者或其他与本研究相关的人员需要加入，也需进行相关培训。

标准操作规程：主要研究者根据需要对本研究制定标准操作规程（SOP）并按照 SOP 的要求进行临床研究管理。本临床研究同时需要遵守医院及科室临床研究相关标准操作规程。

**10 数据处理及保存**

使用CRF表进行原始数据记录和临床研究需要的数据的采集。采用双人数据录入的方式避免和减少数据录入错误。数据管理员进行数据审核，完成数据质疑后锁定数据库，进行统计分析。

**11 统计分析**

**11.1统计分析数据集**

描述统计分析人群定义，未依从研究方案的受试者应重点说明放在什么分析集中，用什么方法处理失访数据。

全分析数据集（Full Analysis Set，FAS）：对所有经随机化分组，并至少完成术后吸氧的全部病例，将其中未能观察到全部治疗过程的病例资料，用最后一次观察数据结转到研究最终结果，对疗效进行意向性治疗分析。FAS人群是本次研究疗效评价的主要人群。

符合方案数据集（Per Protocol Set，PPS）：符合纳入标准并完成治疗方案的病例集合，即符合研究方案，依从性好，完成病例报告表规定填写内容的病例。PPS人群是本次研究疗效评价的次要人群。

安全数据集（Safety Set，SS）：至少完成术后吸氧，且有安全性指标记录的实际数据。不良反应的发生率以安全集的病例数作为分母。

**12 统计分析方法**

采用R studio软件4.2.2版用于统计分析。

**12.1统计描述**

计量资料：采用均数、标准差、中位数、最大值、最小值等进行统计描述。

计数资料：采用频数、构成比、率、95%置信区间描述。

**12.2统计推断**

连续变量：t检验或Mann-Whitney U检验（通过Kolmogorov-Smirnov检验进行检验）

比较分类数据的差异：x2检验或fisher精确检验

统计推断均采用双侧检验（α＝0.05），P<0.05将被认为所检验的差别有统计学意义。

**12.3 统计分析计划**

**12.3.1 基线均衡性检验**

采用 t 检验或 Wilcoxon 秩和检验，χ^2^检验或 Fisher 确切概率方法来比较组间人口学资料及其它基线值的均衡性，对 FAS 集人群进行分析。

**12.3.2 安全性指标分析**

以描述性统计分析为主，列表描述本次试验所发生的不良事件及不良反应。实验室检验结果描述试验前正常但治疗后异常的情况以及发生异常改变时与试验药物的关系。计算吸氧前后生命体征及采用配对 t 检验前后比较。

**13 伦理学原则**

**13.1 伦理委员会审核**

根据赫尔辛基宣言原则以及其他适宜的法规进行本临床研究。研究者和所有临床研究人员根据研究方案进行临床研究。研究者确保所有参与研究的人员具备适当的教育、培训和经验，有资格承担其职责。

临床研究开始前，本研究方案、知情同意书、其他任何给受试者的书面信息和任何受试者招募广告必须经独立伦理委员会批准。批准书必须签署日期，并标明适宜的研究方案、修订版（如有）、知情同意书、所有适宜的招募材料、为受试者提供的书面信息和受试者补偿方案。在研究结束时，研究者通知伦理委员会研究已结束。如果提前终止研究，也必须通知伦理委员会。最后，研究者需要按伦理委员会规定的事件间隔向伦理委员会报告研究进度。

**13.2 知情同意**

在开始任何筛选或其他研究相关的程序前，每位受试者（和/或法定代表，适当情况下）必须自愿签署知情同意书。研究者必须有制定的流程来获取知情同意。尤其是，研究者或被指派需要向每位潜在受试者解释本临床研究，受试者必须自愿在已批准的知情同意书上签署姓名日期。受试者必须有机会向研究者提问，如果当地法规有要求，还可向其他有资质的人员提问。研究者必须向受试者提供一份用其能理解的语言书写的知情同意书副本。知情同意书必须满足所有适宜的当地法律，并告知受试者关于研究目的、程序、要求和限制以及临床研究相关的任何已知风险和潜在收益、赔偿和个人健康信息的保密条款。向受试者告知，参加本研究自愿性质，并向其提供研究期间出现问题或顾虑时的联系人信息。还必须告知受试者，他们的记录可被适当的机构和申办者指定人员查阅。研究者必须保存签字后的知情同意书，并向每位受试者提供副本。

**13.3受试者的信息保密**

研究者必须确保研究期间受试者保持匿名状态，尤其是研究者必须对入组记录保密，仅通过随机号和姓名首字母（或姓名拼音缩写）来识别。

**14 方案的修改**

方案经伦理委员会批准后，若在实施过程中需要修改，由主要研究者撰写“方案修改说明”，并签字，同时需报请伦理委员会批准后方可实施。

**15 试验组织架构**

主办方：成都妇女儿童医院麻醉科

**16 保密及数据安全**

纸质研究资料存放于研究者办公室带锁的抽屉里，研究资料采集后存储于研究者带密码的电脑中。

**17知识产权**

本研究结果及本研究过程中产生的所有知识产权归崔宇所有，在该结果公开发表前，任何人只有在获得崔宇书面同意的前提下，方可发表与本研究相关的论文或其他公开发表的文章、讲演。

**18 参考文献**

1. von Ungern-Sternberg BS, Boda K, Chambers NA, et al. Risk assessment for respiratory complications in paediatric anaesthesia: a prospective cohort study. Lancet. 2010;376(9743):773-783. doi:10.1016/S0140-6736(10)61193-2
2. Li H, Zhang Y, Cai J, Wang H, Wei R. Risk Factors of Hypoxemia in the Postanesthesia Care Unit After General Anesthesia in Children. J Perianesth Nurs. 2023;38(5):799-803. doi:10.1016/j.jopan.2023.02.003
3. Sun Z, Sessler DI, Dalton JE, Devereaux PJ, Shahinyan A, Naylor AJ, et al. Postoperative Hypoxemia Is Common and Persistent: A Prospective Blinded Observational Study. Anesth Analg. 2015;121(3):709-715. doi: 10.1213/ANE.0000000000000836.
4. Kim GS, Song JG, Gwak MS, Yang M. Postoperative outcome in formerly premature infants undergoing herniorrhaphy: comparison of spinal and general anesthesia. J Korean Med Sci. 2003;18(5):691-695. doi:10.3346/jkms.2003.18.5.691
5. Langhan ML, Li FY, Lichtor JL. The impact of capnography monitoring among children and adolescents in the postanesthesia care unit: a randomized controlled trial. Paediatr Anaesth. 2017;27(4):385-393. doi:10.1111/pan.13077
6. Mamie C, Habre W, Delhumeau C, Argiroffo CB, Morabia A. Incidence and risk factors of perioperative respiratory adverse events in children undergoing elective surgery. Paediatr Anaesth. 2004;14(3):218-224. doi:10.1111/j.1460-9592.2004.01169.x
7. Iyer NP, Rotta AT, Essouri S, et al. Association of Extubation Failure Rates With High-Flow Nasal Cannula, Continuous Positive Airway Pressure, and Bilevel Positive Airway Pressure vs Conventional Oxygen Therapy in Infants and Young Children: A Systematic Review and Network Meta-Analysis. JAMA Pediatr. 2023;177(8):774-781. doi:10.1001/jamapediatrics.2023.1478
8. Karedath J, Hatamleh MI, Haseeb R, et al. Comparison of High-Flow Nasal Cannula Versus Conventional Oxygen Therapy After Extubation in Children Undergoing Cardiac Surgery: A Meta-analysis. Cureus. 2023;15(3):e36922. Published 2023 Mar 30. doi:10.7759/cureus.36922
9. Kuitunen I, Uimonen M. Noninvasive respiratory support preventing reintubation after pediatric cardiac surgery-A systematic review. Paediatr Anaesth. 2023. doi:10.1111/pan.14808
10. Testa G, Iodice F, Ricci Z, et al. Comparative evaluation of high-flow nasal cannula and conventional oxygen therapy in paediatric cardiac surgical patients: a randomized controlled trial. Interact Cardiovasc Thorac Surg. 2014;19(3):456-461. doi:10.1093/icvts/ivu171
11. Lee JH, Rehder KJ, Williford L, Cheifetz IM, Turner DA. Use of high flow nasal cannula in critically ill infants, children, and adults: a critical review of the literature. Intensive Care Med. 2013;39(2):247-257. doi:10.1007/s00134-012-2743-5
12. Stevens H, Gallant J, Foster J, Horne D, Krmpotic K. Extubation to High-Flow Nasal Cannula in Infants Following Cardiac Surgery: A Retrospective Cohort Study. J Pediatr Intensive Care. 2021;12(3):167-172. doi:10.1055/s-0041-1730933
13. Huang Y, Zhao J, Hua X, Luo K, Shi Y, Lin Z, Tang J, Feng Z, Mu D; Evidence-Based Medicine Group, Neonatologist Society, Chinese Medical Doctor Association. Guidelines for high-flow nasal cannula oxygen therapy in neonates (2022). J Evid Based Med. 2023;16(3):394-413. doi: 10.1111/jebm.12546.
14. S Rolim D, Galas FRB, Faria LS, Amorim EF, Regenga MM, Troster EJ. Use of Noninvasive Ventilation in Respiratory Failure After Extubation During Postoperative Care in Pediatrics. Pediatr Cardiol. 2020;41(4):729-735. doi: 10.1007/s00246-020-02290-6.

**English translation.**

**Clinical study protocol**

**(Solution version number: V1.0, version date: January 22, 2024)**

**Project name: Comparative evaluation of high-flow nasal cannula and conventional oxygen therapy in young infants: a prospective randomized controlled clinical study**

**Project type: Intervention experiment**

**Project source unit: Chengdu Women and Children's Hospital**

**Project participant: Chengdu Women and Children's Hospital**

**Project Leader (/ Principal investigator) : Yu Cui**

**Department: Department of Anesthesiology**

**catalogue**

1 Research background 4

2 Research objectives 4

2.1 Main research objectives 4

2.2 Secondary research objectives 4

3 Research Design 4

3.1 Sample size calculation 4

3.2 Randomizing 4

3.3 Control 4

3.4 Blinding and unblinding 4

4 Object 5

4.1 Diagnostic Criteria 5

4.2 Inclusion of Standard 5

4.3 Exclusion Criteria 5

4.4 Exit Standard 5

4.5 Shedding and handling Standard 5

4.6 Exclusion of Standard 5

5 Treatment options 6

6 Visit and observe item 6

6.1 General item 6

6.2 Diagnostic and therapeutic indicators 6

6.3 Security Specifications 6

6.4 Inspection Process and Precautions 6

6.5 Patient compliance and withdrawal 7

7 Criteria for efficacy 7

7.1 Assessment of symptoms or signs of efficacy 7

7.2 Evaluation of comprehensive efficacy 7

8 Management and reporting of adverse events 7

8.1 Definition of adverse events 7

8.2 Severity Assessment 7

8.3 Correlation Judgment 8

8.4 Handling of Adverse Events 9

8.5 Recording and reporting of serious adverse events 10

9 Quality Control and Quality Assurance 11

9.1 Quality Control 11

9.2 Quality Assurance 11

10 Data processing and storage 11

11 Statistical analysis 11

12 Statistical analysis methods 12

12.1 Statistical Description 12

12.2 Statistical Inference 13

12.3 Statistical Analysis Plan 13

13 Principles of Ethics 14

13.1 Ethics Committee Review 14

13.2 Informed consent 14

13.3 Confidentiality of subject's information 15

14 Modification of the scheme 16

15 Test organizational structure 16

16 Confidentiality and data security 16

17 Insurance and compensation 16

18 Intellectual Property 16

19 References (Please attach relevant references) 16

**Scheme summary**

It was performed in the main operating room of Chengdu Women and Children's Central Hospital from March 10, 2024 to March 10, 2026. 384 children younger than 1 year old will be included in the study. The research team will evaluate patients before surgery and obtain written informed consent from each candidate's family. Demographic data and medical examinations will be collected from medical records. Patients were randomly divided into conventional oxygen therapy group and nasal high flow oxygen inhalation group. Computer-generated random coding was used immediately before anesthesia, using sealed opaque envelopes for random grouping. Given the nature of the research intervention, a completely blind approach is not possible. However, the assignment of groups to the investigators involved in the post-operative assessment was not known. All patients fasted solid food for more than 8 hours and sugar water for 2 hours before surgery. In the operating room, ECG, pulse oximetry, noninvasive blood pressure, and mean arterial pressure were continuously monitored. All patients were treated with general anesthesia. After the operation, they were sent to the post-anesthesia care unit for resuscitation, and then the tracheal catheter was removed for routine oxygen therapy or nasal high-flow oxygen inhalation. The heart rate and SPO2 were recorded in different periods.

result

The primary outcome was the number of children with SpO2 ≤ 95% after oxygen therapy

Mild upper respiratory tract obstruction can be alleviated by changing the sleeping position or gently lifting the jaw of the child, and severe respiratory depression, including the use of oropharyngeal airway and laryngeal mask, can be alleviated, the proportion of postoperative tracheal intubation, the proportion of postoperative unplanned transfer to ICU, the duration of postoperative oxygen inhalation, the duration of postoperative recovery room stay, and the duration of postoperative feeding. The number of children with nasal mucosal injury was assessed as a secondary outcome.

**Glossary of abbreviations**

Not involve

**1.Background**

Postoperative hypoxemia after general anesthesia is very common, ranged from 4%~ 57%. To our knowledge, it occurs more often in young infants than in older children or adults. The risk factors associated with postoperative hypoxemia in children included age, body weight, surgical type, anesthesia method, insufficient oxygen supplement during transportation, and comorbidity (i.e., obstructive sleep apnea syndrome).

Young infants, with premature lung, or even hemodynamically unstable, might increase the risk of postoperative hypoxemia. Traditionally, convention oxygen therapy (COT) was routinely used in postanesthesia care unit (PACU) to decrease postoperative pulmonary complications. However, although oxygen supplement was administrated, the incidence postoperative hypoxemia in newborn and infant was still as high as 3.8%. Thus, it is urgent to explore strategies to decrease the incidence of hypoxemia.

The use of “high flow nasal cannula” (HFNC), a relatively new mode of respiratory support, is increasingly being used in infants [Iyer, 2023]. It is mainly applied in the field of intensive care unit (ICU) and after extubation in children undergoing cardiac surgery. The advantages after cardiac surgery still under-determined. A meta-analysis had reported that compared with COT, HFNC significantly increased PaO2 and the ratio of PaO2 to FiO2, and decreased PaCO2 after cardiac surgery; whereas, Kuitunen et al. did not find clear evidence of a difference in reintubation rates and length of PICU stay between different noninvasive ventilation strategies. In our hospital, more than 10 thousand pediatric non-cardiac surgeries are performed per year, of which 10% are infants. For infants, their oxygen reserve is poor, and they are more prone to develop desaturation after surgery. Whether HFNC is more effective than COT to ensure oxygen supply after non-cardiac surgery never had been investigated. Thus, we intent to conduct a randomized clinical trial to investigate the advantages of HFNC in lower the risk of desaturation after general anesthesia in infants. We hypothesized that prophylactic use of HFNC after non-cardiac surgery in infants would reduce the incidence of post-anesthesia hypoxemia and could be also adapted as first-line oxygen therapy after non-cardiac surgery.

**2 Research Objectives**

To investigate the advantages of HFNC in reducing the risk of oxygenation reduction after general anesthesia in infants

**3 Experimental design**

Prospective randomized controlled clinical study

**3.1** **Sample size**

The current study is powered by the primary outcome. The sample size in this prospective study is calculated according to the authors’ pilot study with the desaturation rate of 20.5% and 10% in the COT group and the HFNC group, respectively, with a standard deviation of 0.2. To achieve an 80% statistical power with an a error of 0.05, the number of patients was calculated to be 174 for each group. Considering 10% of sample drop, 192 patients were enrolled in each group.

**3.2 Randomization**

Patients were randomly divided into COT group and the HFNC group. Computer-generated random coding was used immediately before anesthesia, using sealed opaque envelopes for random grouping.

**3.3 Control**

Use regular oxygen.

**3.4 Blinding method and unblinding**

This is a randomized, single-blind controlled clinical trial. However, the researcher analyzing the data will be blinded to the groups’ assignment.

**4 Patients**

**4.1 Inclusion criteria**

Infants under the age of 12 months who are scheduled to undergo elective non-cardiac surgery; American Society of Anesthesiologists classification Ⅰ~Ⅱ; Endotracheal tube is removed in the PACU. Family members of the child volunteered to join and signed a written informed consent.

**4.2 Exclusion criteria**

Patients with cardiac/hepatic/renal dysfunction or congenital airway malformation; Patients with any allergic history of anesthetic;SpO2<95% in room air before anesthesia. Their guardians refuse to participate.

**4.3 Exit Standards**

The child's guardian refused to participate.

**4.4 Shedding and Treatment of shedding**

① Children with nasal bleeding can not use high-flow oxygen; Guardian of child missing visit. (2) Treatment of shedding: The researcher should contact the subject as much as possible to ask the reason; For those who withdraw from the study due to allergic reactions, adverse events, or ineffective treatment, the researcher shall properly treat the subjects according to the actual situation to protect the rights and interests of the subjects.

**4.5 Exclusion Criteria**

Serious protocol violations, such as misinclusion of subjects (violation of inclusion/exclusion criteria), etc.

**5 Treatment Plan**

Experimental group: postoperative nasal high flow oxygen inhalation

Control group: postoperative conventional oxygen therapy

**6. Visit and observe project**

**6.1 General Items**

Including vital signs, demographics, family history, past history, combined disease and treatment history, current drug use history and trial study related symptoms and signs.

**6.2 Diagnostic and therapeutic indicators**

the rate of desaturation post-extubation. which is defined as SpO2 ≤ 95%.;mild upper airway obstruction which can be improved by changing the posture; severe respiratory depression needs airway management including laryngeal mask ventilation, or placement of other airway device, the rate of tracheal reintubation; duration of oxygen therapy; length of PACU stay; the time to reach full enteral feeding, and the rate of, nasal injury.

**6.3 Security Specifications**

Expected or unexpected adverse events。

**6.4 Inspection Process and Precautions**

| **time**  **step** | | Screening period | Screening period | Screening period |
| --- | --- | --- | --- | --- |
|  | Sign informed consent |  | × | × |
| Clinical data | Basic data of subjects |  |  |  |
| Inclusion and exclusion criteria | |  | × | × |
| Subject Visit | Visit 1 (during extubation) | × |  |  |
|  | Visit 2 (72h after extubation) | × |  |  |
|  | Visit 3 (upon discharge) | × |  |  |
| Adverse event and device defect records (not required) | | × |  |  |
| Scheme deviation record (not required) | | × |  |  |
| Test completion | | × | × |  |
| Fill in CRF | |  |  |  |

**6.5 Patient compliance**

Gradually increase the oxygen absorption flow, and stop increasing the oxygen absorption flow when the patient is uncomfortable.

**7 Criteria for evaluation of curative effect**

**7.1 Assessment of symptoms or signs of efficacy**

SpO2 occurred after oxygen inhalation.

**8 Handling and reporting of adverse events**

**8.1 Definition of adverse events**

Adverse Event (AE) : An adverse medical event that occurs after a patient or clinical study subject receives the investigational treatment, but is not necessarily causally related to the treatment. Therefore, an adverse event may be an adverse and unexpected sign (e.g. including abnormal laboratory tests), symptom, or disease that is time-related to treatment, regardless of whether there is a causal relationship with the treatment administered.

Serious Adverse Event (SAE) refers to an event that occurs during a clinical study that requires hospitalization, prolongation of hospital stay, disability, impact on work ability, life-threatening or death, or congenital malformation.

**8.2 Severity assessment**

Adverse events will be evaluated with reference to the NCI-CTCAE (version 5.0). If the severity of an adverse event is not specified in the guidelines, investigators may evaluate it according to the general definition of level 1 to 5, combined with medical judgment. General classification of adverse events:

Level 1: mild; Asymptomatic or mild; Clinical or diagnostic findings only; No treatment required.

Level 2: moderate; Require minor, local or non-invasive treatment; Age-appropriate restriction in instrumental activities of daily living *.

Level 3: serious or medically important but not immediately life-threatening; Resulting in hospitalization or prolonged hospitalization; To become disabled; Limitations in activities of daily living **.

Level 4: life-threatening; Need urgent medical attention.

Level 5: Death related to AE.

Semicolon (;) used in level descriptions The value is either.

* Instrumental activities of daily living refer to cooking, buying clothes, using the telephone, managing money, etc.

** Self-rational daily living activities refer to bathing, dressing, eating, washing, taking drugs, etc., and are not bedridden.

**8.3 Correlation judgment**

The relationship between adverse events and the conduct of the study was classified as positive, very likely, possibly relevant, possibly unrelated, and definitely unrelated.

Definitely related: Adverse events conform to the common types of adverse events of treatment, their occurrence has a reasonable time relationship with treatment, the adverse events disappear after treatment is stopped, and the adverse events are not related to co-treatment (medication or other therapeutic procedures) or non-therapeutic factors (primary disease, complications, food, environment, etc.).

Likely to be relevant: Adverse events conform to common types of treatment adverse events, their occurrence has a reasonable time relationship with treatment, adverse events are significantly alleviated after treatment is discontinued, and adverse events are not related to combination therapy (medication or other therapeutic procedures) or non-therapeutic factors (primary disease, complications, food, environment, etc.).

May be relevant: The adverse event is consistent with the common type of treatment adverse event, its occurrence has a reasonable time relationship with treatment, the adverse event may resolve after treatment discontinuation, and the adverse event may be explained by combination of therapeutic or non-therapeutic factors.

May not be relevant: Adverse events had a reasonable time relationship with treatment, but did not conform to the common types of adverse reactions, and the remission of adverse events was not obvious after treatment was stopped; A combination of therapeutic or non-therapeutic factors can explain the adverse event, and the adverse event may be alleviated after removal of these factors.

Definitely not relevant: Adverse events did not have a reasonable time relationship with treatment, did not conform to the common types of adverse reactions of treatment, and adverse events did not relieve after treatment was stopped; The adverse events were definitely related to the combination of therapeutic or non-therapeutic factors, and the adverse events were significantly alleviated or disappeared after the removal of such factors.

**8.4 Management of adverse events**

**8.4.1 Record of adverse events**

At each visit, all adverse events observed or mentioned by subjects were recorded by the investigator in the source data. Records include the following: event description, severity, start date and end date, outcome, relationship with clinical study treatment, whether action was taken on the event, severity evaluation.

**8.4.2 Treatment and follow-up**

The investigator should take appropriate measures according to the adverse events to ensure the health and safety of the subjects; The profession shall ensure the supply of relevant rescue equipment and drugs to ensure that appropriate treatment can be given in an emergency.

Adverse events need to be followed by the investigator until they disappear/return to baseline levels or final results are known. The tracking results should also be recorded in the original data.

**8.5 Record and report of serious adverse events**

Saes that occurred between the time the subject signed the informed consent and the last follow-up were collected and recorded. In the event of an SAE, researchers should complete and sign the Serious Adverse Event Report Form in as much detail as possible. The investigators report to the Clinical Research Administration (Science and Education Department) and the hospital Ethics committee within 24 hours of receiving SAE information. In accordance with the Clinical Research management Department and ethics committee of the relevant management practices.

**9 Quality control and quality assurance**

**9.1 Quality Control**

Investigator Authorization: All investigators, only after receiving training and obtaining authorization from the principal investigator, may enter clinical research and operate within the scope of authorization.

Research process quality control: Project team/professional group quality control personnel, clinical research management department quality manager to conduct quality control of clinical research process, guide researchers in accordance with the research protocol and clinical research management requirements of clinical research.

**9.2 Quality Assurance**

Investigator training: Before the start of the study, the principal investigator shall train all the researchers participating in the study and other personnel related to the study on the management documents, Sops and the study protocol related to the hospital IIT clinical study management; And the regulation of the monitoring standards of the main therapeutic efficacy measurement indicators related to the study. During the research process, if new researchers or other personnel related to the research need to join, relevant training is also required.

Standard Operating Procedures: The principal investigator formulated standard operating procedures (Sops) for this study as required and conducted clinical study management in accordance with the requirements of the Sops. This clinical study also needs to comply with the hospital and department clinical research related standard operating procedures.

**10 10 Data processing and storage**

CRF table is used for original data recording and collection of data required for clinical study. Use two-person data entry method to avoid and reduce data entry errors. The data administrator conducts data audit, locks the database after data questioning, and performs statistical analysis.

**11 Statistical Analysis**

**11.1 Statistical analysis data set**

Describe the statistical analysis population definition. Subjects who do not comply with the study protocol should highlight what analysis set to place in and what method to use to process the lost follow-up data.

Full Analysis Set (FAS) : All cases randomized and at least completed postoperative oxygen inhalation were randomized, and the data of those cases in which the full course of treatment could not be observed were carried forward to the final results of the study with the last observation data, and the therapeutic effect was analyzed intentionally. FAS population was the main population for efficacy evaluation in this study.

Per Protocol Set (PPS) : The set of cases that meet the inclusion criteria and complete the treatment plan, that is, the cases that meet the study plan, have good compliance, and complete the contents specified in the case report form. PPS population was the secondary population for efficacy evaluation in this study.

Safety Set (SS) : Actual data for which at least postoperative oxygen inhalation has been completed and safety indicators have been recorded. The incidence of adverse events was determined by the number of cases in the safe

**12**.**method of Statistical Analysis**

The R studio software, version 4.2.2, will be used for statistical analysis.

**12.1 Statistical Description**

Measurement data: The mean, standard deviation, median, maximum and minimum values are used for statistical description.

Counting data: Frequency, component ratio, rate, 95% confidence interval were used to describe.

**12.2 Statistical Inference**

Continuous variable: t test or Mann-Whitney U test

Compare differences in classified data: χ2 test or fisher exact test

Bilateral test (α = 0.05) was used for all statistical inferences, and P<0.05 would be considered statistically sign

**12.3 Statistical analysis plan**

**12.3.1 Baseline Balance Check**

The T-test or Wilcoxon rank sum test, χ2 test or Fisher exact probability method were used to compare the inter-group demographic data and the equilibrium of other baseline values for the FAS set population.

**12.3.2 Security Indicator Analysis**

Based on descriptive statistical analysis, the adverse events and adverse reactions occurred in this study were listed. Laboratory test results describe what is normal before the trial but abnormal after treatment and how the abnormal changes relate to the test drug. The vital signs before and after oxygen inhalation were calculated and compared with those before and after paired T-test.

**13 Ethical Principles**

**13.1 Ethics Committee review**

This clinical study was conducted in accordance with the principles of the Declaration of Helsinki and other appropriate regulations. The investigator and all clinical investigators conduct clinical studies according to the research protocol. The Investigator ensures that all research participants have the appropriate education, training, and experience to be qualified to perform their duties.

This study protocol, informed consent, any other written information to subjects and any recruitment advertisement for subjects must be approved by an independent Ethics Committee prior to the commencement of the clinical study. The approval letter must be dated and indicate the appropriate study protocol, revision (if any), informed consent, all appropriate recruitment materials, written information for subjects, and subject compensation options. At the end of the study, the investigator informed the Ethics Committee that the study was closed. The Ethics Committee must also be informed if the study is terminated prematurely. Finally, the investigator is required to report the progress of the research to the Ethics Committee at the event intervals specified by the Ethics Committee.

**13.2 Informed consent**

Before commencing any screening or other study-related procedures, each subject (and/or legal representative, where appropriate) must voluntarily sign an informed consent form. Researchers must have procedures in place to obtain informed consent. In particular, the investigator may be assigned to explain the clinical study to each potential subject, who must voluntarily sign and date the approved informed consent form. Subjects must have the opportunity to ask questions of the investigator and, if required by local regulations, other qualified personnel. The investigator must provide the subject with a copy of the informed consent written in a language they understand. Informed consent must meet all appropriate local laws and inform subjects of the study purpose, procedures, requirements, and limitations, as well as the confidentiality of any known risks and potential benefits associated with the clinical study, compensation, and personal health information. Inform subjects that participation in the study is voluntary and provide them with contact information for questions or concerns during the study. Subjects must also be informed that their records can be accessed by appropriate institutions and sponsor designators. The investigator must keep the signed informed consent and provide a copy to each subject.

**13.3 The subject's information shall be kept confidential**

The investigator must ensure that participants remain anonymous during the study, and in particular the investigator must keep enrollment records confidential and identify them only by random numbers and initials (or initials in pinyin).

**14 Modification of scheme**

After the program is approved by the Ethics Committee, if it needs to be modified during the implementation process, the main researcher shall write and sign the "Modification Description of the program" and submit it to the Ethics Committee for approval before it can be implemented.

**15 Experimental organization structure**

Sponsor: Department of Anesthesiology, Chengdu Women's and Children's Hospital

**16 Confidentiality and data security**

Paper research data is stored in a locked drawer in the researcher's office, and the data is collected and stored in the researcher's computer with a password.

**17 Intellectual Property**

The results of this research and all intellectual property rights generated in the process of this research belong to Yu Cui. Before the public publication of the results, anyone can publish the paper or other publicly published articles or lectures related to this research only with the written consent of Yu Cui.

**18 Reference**

1. von Ungern-Sternberg BS, Boda K, Chambers NA, et al. Risk assessment for respiratory complications in paediatric anaesthesia: a prospective cohort study. Lancet. 2010;376(9743):773-783. doi:10.1016/S0140-6736(10)61193-2

2. Li H, Zhang Y, Cai J, Wang H, Wei R. Risk Factors of Hypoxemia in the Postanesthesia Care Unit After General Anesthesia in Children. J Perianesth Nurs. 2023;38(5):799-803. doi:10.1016/j.jopan.2023.02.003

3. Sun Z, Sessler DI, Dalton JE, Devereaux PJ, Shahinyan A, Naylor AJ, et al. Postoperative Hypoxemia Is Common and Persistent: A Prospective Blinded Observational Study. Anesth Analg. 2015;121(3):709-715. doi: 10.1213/ANE.0000000000000836.

4. Kim GS, Song JG, Gwak MS, Yang M. Postoperative outcome in formerly premature infants undergoing herniorrhaphy: comparison of spinal and general anesthesia. J Korean Med Sci. 2003;18(5):691-695. doi:10.3346/jkms.2003.18.5.691

5. Langhan ML, Li FY, Lichtor JL. The impact of capnography monitoring among children and adolescents in the postanesthesia care unit: a randomized controlled trial. Paediatr Anaesth. 2017;27(4):385-393. doi:10.1111/pan.13077

6. Mamie C, Habre W, Delhumeau C, Argiroffo CB, Morabia A. Incidence and risk factors of perioperative respiratory adverse events in children undergoing elective surgery. Paediatr Anaesth. 2004;14(3):218-224. doi:10.1111/j.1460-9592.2004.01169.x

7. Iyer NP, Rotta AT, Essouri S, et al. Association of Extubation Failure Rates With High-Flow Nasal Cannula, Continuous Positive Airway Pressure, and Bilevel Positive Airway Pressure vs Conventional Oxygen Therapy in Infants and Young Children: A Systematic Review and Network Meta-Analysis. JAMA Pediatr. 2023;177(8):774-781. doi:10.1001/jamapediatrics.2023.1478

8. Karedath J, Hatamleh MI, Haseeb R, et al. Comparison of High-Flow Nasal Cannula Versus Conventional Oxygen Therapy After Extubation in Children Undergoing Cardiac Surgery: A Meta-analysis. Cureus. 2023;15(3):e36922. Published 2023 Mar 30. doi:10.7759/cureus.36922

9. Kuitunen I, Uimonen M. Noninvasive respiratory support preventing reintubation after pediatric cardiac surgery-A systematic review. Paediatr Anaesth. 2023. doi:10.1111/pan.14808

10. Testa G, Iodice F, Ricci Z, et al. Comparative evaluation of high-flow nasal cannula and conventional oxygen therapy in paediatric cardiac surgical patients: a randomized controlled trial. Interact Cardiovasc Thorac Surg. 2014;19(3):456-461. doi:10.1093/icvts/ivu171

11. Lee JH, Rehder KJ, Williford L, Cheifetz IM, Turner DA. Use of high flow nasal cannula in critically ill infants, children, and adults: a critical review of the literature. Intensive Care Med. 2013;39(2):247-257. doi:10.1007/s00134-012-2743-5

12. Stevens H, Gallant J, Foster J, Horne D, Krmpotic K. Extubation to High-Flow Nasal Cannula in Infants Following Cardiac Surgery: A Retrospective Cohort Study. J Pediatr Intensive Care. 2021;12(3):167-172. doi:10.1055/s-0041-1730933

13. Huang Y, Zhao J, Hua X, Luo K, Shi Y, Lin Z, Tang J, Feng Z, Mu D; Evidence-Based Medicine Group, Neonatologist Society, Chinese Medical Doctor Association. Guidelines for high-flow nasal cannula oxygen therapy in neonates (2022). J Evid Based Med. 2023;16(3):394-413. doi: 10.1111/jebm.12546.

14. S Rolim D, Galas FRB, Faria LS, Amorim EF, Regenga MM, Troster EJ. Use of Noninvasive Ventilation in Respiratory Failure After Extubation During Postoperative Care in Pediatrics. Pediatr Cardiol. 2020;41(4):729-735. doi: 10.1007/s00246-020-02290-6.
